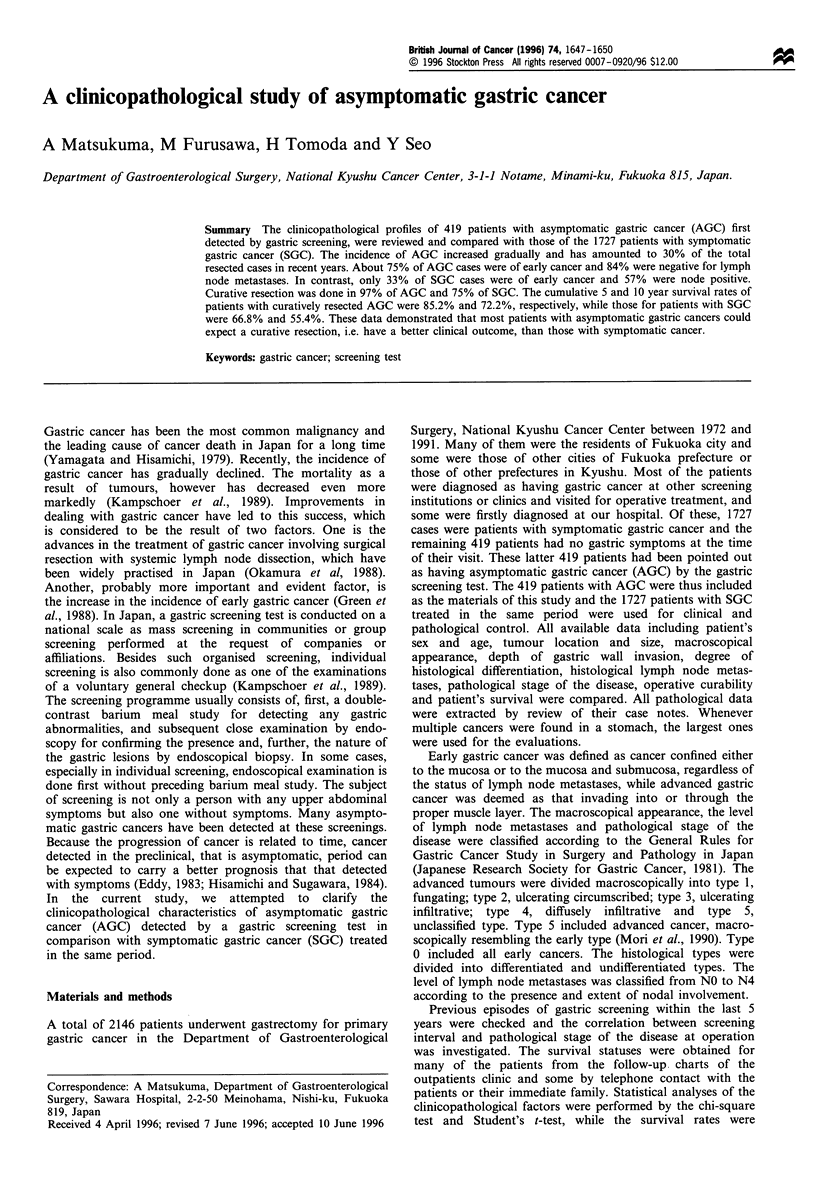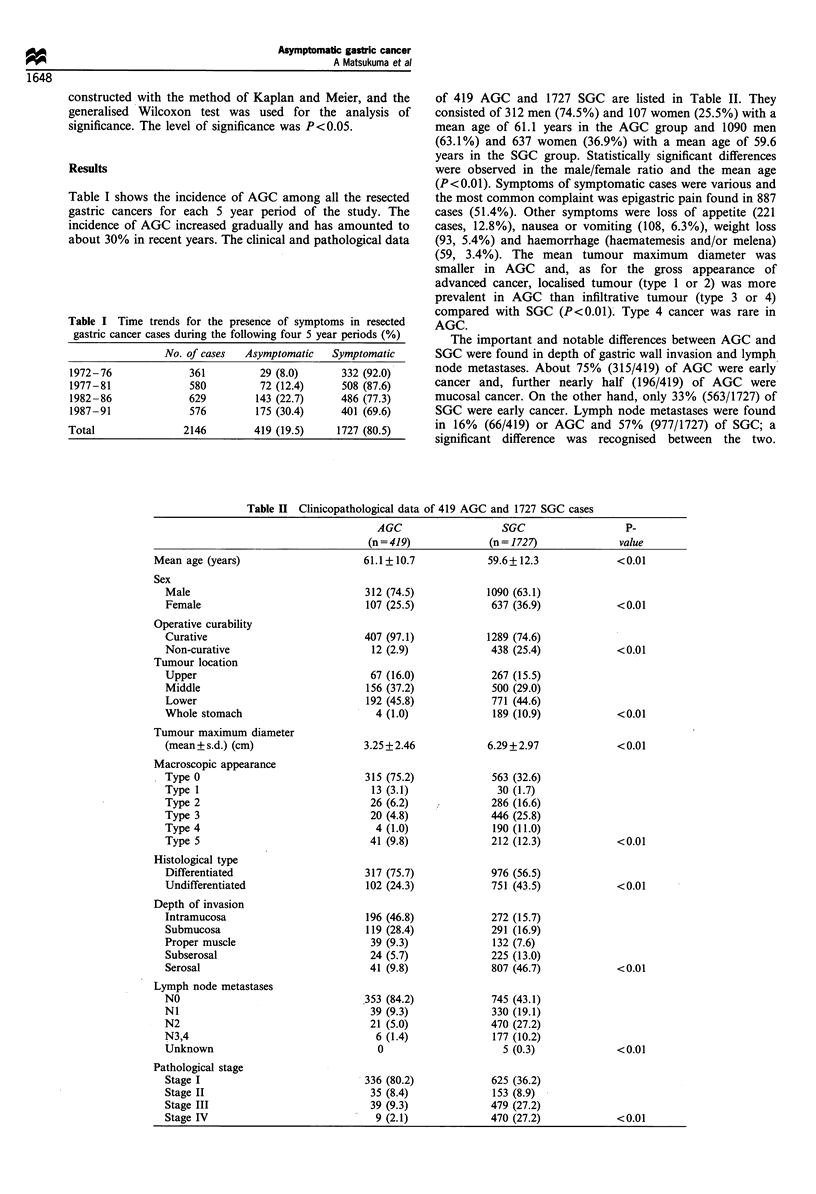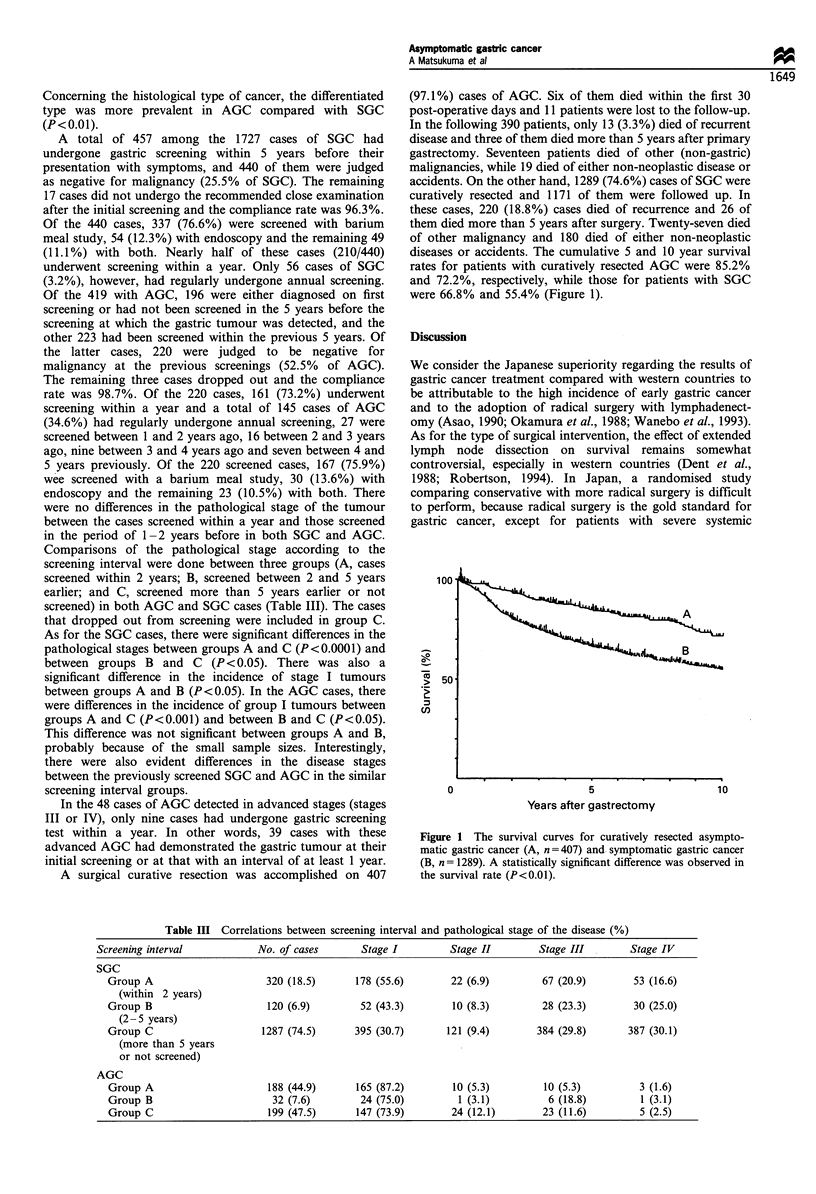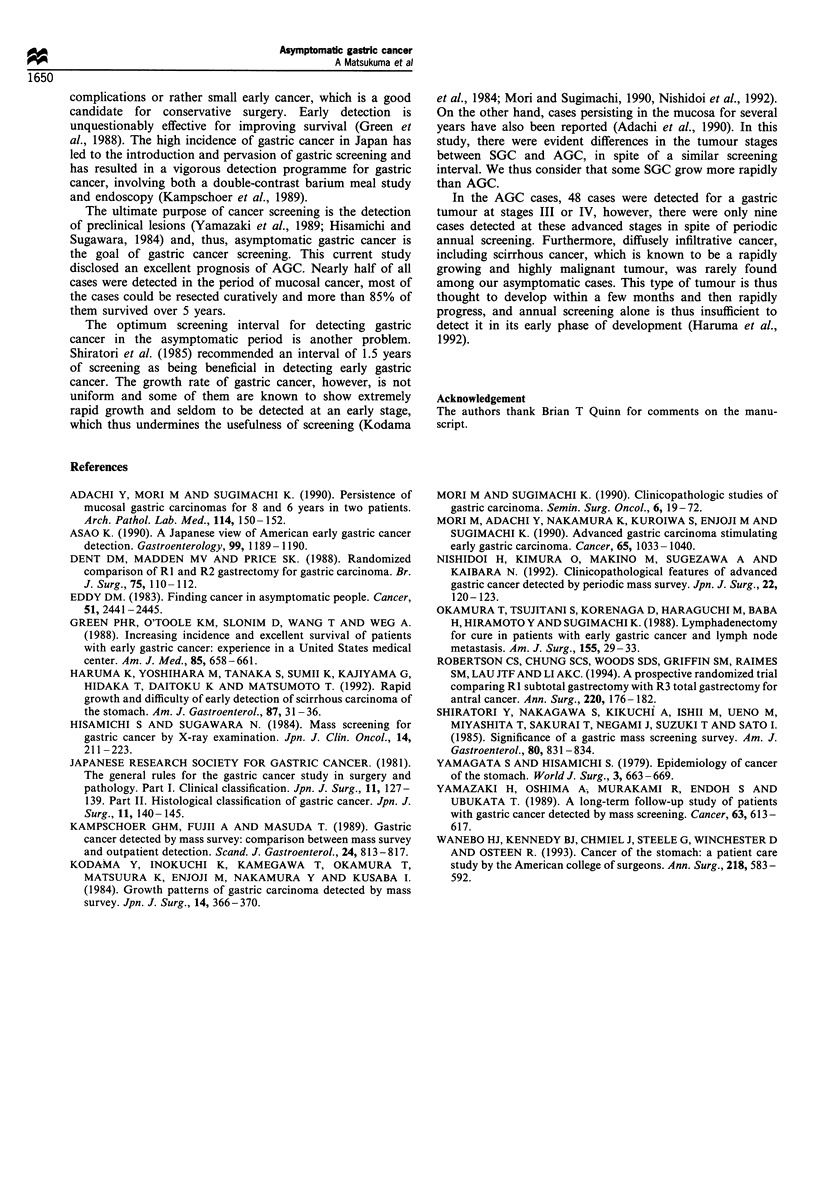# A clinicopathological study of asymptomatic gastric cancer.

**DOI:** 10.1038/bjc.1996.603

**Published:** 1996-11

**Authors:** A. Matsukuma, M. Furusawa, H. Tomoda, Y. Seo

**Affiliations:** Department of Gastroenterological Surgery, National Kyushu Cancer Center, Fukuoka, Japan.

## Abstract

The clinicopathological profiles of 419 patients with asymptomatic gastric cancer (AGC) first detected by gastric screening, were reviewed and compared with those of the 1727 patients with symptomatic gastric cancer (SGC). The incidence of AGC increased gradually and has amounted to 30% of the total resected cases in recent years. About 75% of AGC cases were of early cancer and 84% were negative for lymph node metastases. In contrast, only 33% of SGC cases were of early cancer and 57% were node positive. Curative resection was done in 97% of AGC and 75% of SGC. The cumulative 5 and 10 year survival rates of patients with curatively resected AGC were 85.2% and 72.2%, respectively, while those for patients with SGC were 66.8% and 55.4%. These data demonstrated that most patients with asymptomatic gastric cancers could expect a curative resection, i.e. have a better clinical outcome, than those with symptomatic cancer.


British Journal of Cancer (1996) 74, 1647-1650
© 1996 Stockton Press All rights reserved 0007-0920/96 $12.00

### A clinicopathological study of asymptomatic gastric cancer

A Matsukuma, M Furusawa, H Tomoda and Y Seo

*Department of Gastroenterological Surgery, National Kyushu Cancer Center, 3-1-1 Notame, Minami-ku, Fukuoka 815, Japan.*

**Summary** The clinicopathological profiles of 419 patients with asymptomatic gastric cancer (AGC) first detected by gastric screening, were reviewed and compared with those of the 1727 patients with symptomatic gastric cancer (SGC). The incidence of AGC increased gradually and has amounted to 30% of the total resected cases in recent years. About 75% of AGC cases were of early cancer and 84% were negative for lymph node metastases. In contrast, only 33% of SGC cases were of early cancer and 57% were node positive. Curative resection was done in 97% of AGC and 75% of SGC. The cumulative 5 and 10 year survival rates of patients with curatively resected AGC were 85.2% and 72.2%, respectively, while those for patients with SGC were 66.8% and 55.4%. These data demonstrated that most patients with asymptomatic gastric cancers could expect a curative resection, i.e. have a better clinical outcome, than those with symptomatic cancer.

**Keywords:** gastric cancer; screening test

Gastric cancer has been the most common malignancy and the leading cause of cancer death in Japan for a long time (Yamagata and Hisamichi, 1979). Recently, the incidence of gastric cancer has gradually declined. The mortality as a result of tumours, however has decreased even more markedly (Kampschoer *et al.*, 1989). Improvements in dealing with gastric cancer have led to this success, which is considered to be the result of two factors. One is the advances in the treatment of gastric cancer involving surgical resection with systemic lymph node dissection, which have been widely practised in Japan (Okamura *et al*, 1988). Another, probably more important and evident factor, is the increase in the incidence of early gastric cancer (Green *et al.*, 1988). In Japan, a gastric screening test is conducted on a national scale as mass screening in communities or group screening performed at the request of companies or affiliations. Besides such organised screening, individual screening is also commonly done as one of the examinations of a voluntary general checkup (Kampschoer *et al.*, 1989). The screening programme usually consists of, first, a double-contrast barium meal study for detecting any gastric abnormalities, and subsequent close examination by endoscopy for confirming the presence and, further, the nature of the gastric lesions by endoscopical biopsy. In some cases, especially in individual screening, endoscopical examination is done first without preceding barium meal study. The subject of screening is not only a person with any upper abdominal symptoms but also one without symptoms. Many asymptomatic gastric cancers have been detected at these screenings. Because the progression of cancer is related to time, cancer detected in the preclinical, that is asymptomatic, period can be expected to carry a better prognosis that that detected with symptoms (Eddy, 1983; Hisamichi and Sugawara, 1984). In the current study, we attempted to clarify the clinicopathological characteristics of asymptomatic gastric cancer (AGC) detected by a gastric screening test in comparison with symptomatic gastric cancer (SGC) treated in the same period.

### Materials and methods

A total of 2146 patients underwent gastrectomy for primary gastric cancer in the Department of Gastroenterological Surgery, National Kyushu Cancer Center between 1972 and 1991. Many of them were the residents of Fukuoka city and some were those of other cities of Fukuoka prefecture or those of other prefectures in Kyushu. Most of the patients were diagnosed as having gastric cancer at other screening institutions or clinics and visited for operative treatment, and some were firstly diagnosed at our hospital. Of these, 1727 cases were patients with symptomatic gastric cancer and the remaining 419 patients had no gastric symptoms at the time of their visit. These latter 419 patients had been pointed out as having asymptomatic gastric cancer (AGC) by the gastric screening test. The 419 patients with AGC were thus included as the materials of this study and the 1727 patients with SGC treated in the same period were used for clinical and pathological control. All available data including patient's sex and age, tumour location and size, macroscopical appearance, depth of gastric wall invasion, degree of histological differentiation, histological lymph node metastases, pathological stage of the disease, operative curability and patient's survival were compared. All pathological data were extracted by review of their case notes. Whenever multiple cancers were found in a stomach, the largest ones were used for the evaluations.

Early gastric cancer was defined as cancer confined either to the mucosa or to the mucosa and submucosa, regardless of the status of lymph node metastases, while advanced gastric cancer was deemed as that invading into or through the proper muscle layer. The macroscopical appearance, the level of lymph node metastases and pathological stage of the disease were classified according to the General Rules for Gastric Cancer Study in Surgery and Pathology in Japan (Japanese Research Society for Gastric Cancer, 1981). The advanced tumours were divided macroscopically into type 1, fungating; type 2, ulcerating circumscribed; type 3, ulcerating infiltrative; type 4, diffusely infiltrative and type 5, unclassified type. Type 5 included advanced cancer, macroscopically resembling the early type (Mori *et al.*, 1990). Type 0 included all early cancers. The histological types were divided into differentiated and undifferentiated types. The level of lymph node metastases was classified from N0 to N4 according to the presence and extent of nodal involvement.

Previous episodes of gastric screening within the last 5 years were checked and the correlation between screening interval and pathological stage of the disease at operation was investigated. The survival statuses were obtained for many of the patients from the follow-up charts of the outpatients clinic and some by telephone contact with the patients or their immediate family. Statistical analyses of the clinicopathological factors were performed by the chi-square test and Student's *t*-test, while the survival rates were

Correspondence: A Matsukuma, Department of Gastroenterological Surgery, Sawara Hospital, 2-2-50 Meinohama, Nishi-ku, Fukuoka 819, Japan
Received 4 April 1996; revised 7 June 1996; accepted 10 June 1996

constructed with the method of Kaplan and Meier, and the generalised Wilcoxon test was used for the analysis of significance. The level of significance was $P<0.05$.

### Results

Table I shows the incidence of AGC among all the resected gastric cancers for each 5 year period of the study. The incidence of AGC increased gradually and has amounted to about 30% in recent years. The clinical and pathological data of 419 AGC and 1727 SGC are listed in Table II. They consisted of 312 men (74.5%) and 107 women (25.5%) with a mean age of 61.1 years in the AGC group and 1090 men (63.1%) and 637 women (36.9%) with a mean age of 59.6 years in the SGC group. Statistically significant differences were observed in the male/female ratio and the mean age ($P<0.01$). Symptoms of symptomatic cases were various and the most common complaint was epigastric pain found in 887 cases (51.4%). Other symptoms were loss of appetite (221 cases, 12.8%), nausea or vomiting (108, 6.3%), weight loss (93, 5.4%) and haemorrhage (haematemesis and/or melena) (59, 3.4%). The mean tumour maximum diameter was smaller in AGC and, as for the gross appearance of advanced cancer, localised tumour (type 1 or 2) was more prevalent in AGC than infiltrative tumour (type 3 or 4) compared with SGC ($P<0.01$). Type 4 cancer was rare in AGC.

The important and notable differences between AGC and SGC were found in depth of gastric wall invasion and lymph node metastases. About 75% (315/419) of AGC were early cancer and, further nearly half (196/419) of AGC were mucosal cancer. On the other hand, only 33% (563/1727) of SGC were early cancer. Lymph node metastases were found in 16% (66/419) or AGC and 57% (977/1727) of SGC; a significant difference was recognised between the two.

**Table I** Time trends for the presence of symptoms in resected gastric cancer cases during the following four 5 year periods (%)

| | *No. of cases* | *Asymptomatic* | *Symptomatic* |
|---|---|---|---|
| 1972–76 | 361 | 29 (8.0) | 332 (92.0) |
| 1977–81 | 580 | 72 (12.4) | 508 (87.6) |
| 1982–86 | 629 | 143 (22.7) | 486 (77.3) |
| 1987–91 | 576 | 175 (30.4) | 401 (69.6) |
| Total | 2146 | 419 (19.5) | 1727 (80.5) |

**Table II** Clinicopathological data of 419 AGC and 1727 SGC cases

| | *AGC* (n=*419*) | *SGC* (n=*1727*) | P-*value* |
|---|---|---|---|
| Mean age (years) | 61.1±10.7 | 59.6±12.3 | <0.01 |
| Sex | | | |
| Male | 312 (74.5) | 1090 (63.1) | |
| Female | 107 (25.5) | 637 (36.9) | <0.01 |
| Operative curability | | | |
| Curative | 407 (97.1) | 1289 (74.6) | |
| Non-curative | 12 (2.9) | 438 (25.4) | <0.01 |
| Tumour location | | | |
| Upper | 67 (16.0) | 267 (15.5) | |
| Middle | 156 (37.2) | 500 (29.0) | |
| Lower | 192 (45.8) | 771 (44.6) | |
| Whole stomach | 4 (1.0) | 189 (10.9) | <0.01 |
| Tumour maximum diameter (mean±s.d.) (cm) | 3.25±2.46 | 6.29±2.97 | <0.01 |
| Macroscopic appearance | | | |
| Type 0 | 315 (75.2) | 563 (32.6) | |
| Type 1 | 13 (3.1) | 30 (1.7) | |
| Type 2 | 26 (6.2) | 286 (16.6) | |
| Type 3 | 20 (4.8) | 446 (25.8) | |
| Type 4 | 4 (1.0) | 190 (11.0) | |
| Type 5 | 41 (9.8) | 212 (12.3) | <0.01 |
| Histological type | | | |
| Differentiated | 317 (75.7) | 976 (56.5) | |
| Undifferentiated | 102 (24.3) | 751 (43.5) | <0.01 |
| Depth of invasion | | | |
| Intramucosa | 196 (46.8) | 272 (15.7) | |
| Submucosa | 119 (28.4) | 291 (16.9) | |
| Proper muscle | 39 (9.3) | 132 (7.6) | |
| Subserosal | 24 (5.7) | 225 (13.0) | |
| Serosal | 41 (9.8) | 807 (46.7) | <0.01 |
| Lymph node metastases | | | |
| N0 | 353 (84.2) | 745 (43.1) | |
| N1 | 39 (9.3) | 330 (19.1) | |
| N2 | 21 (5.0) | 470 (27.2) | |
| N3,4 | 6 (1.4) | 177 (10.2) | |
| Unknown | 0 | 5 (0.3) | <0.01 |
| Pathological stage | | | |
| Stage I | 336 (80.2) | 625 (36.2) | |
| Stage II | 35 (8.4) | 153 (8.9) | |
| Stage III | 39 (9.3) | 479 (27.2) | |
| Stage IV | 9 (2.1) | 470 (27.2) | <0.01 |

Concerning the histological type of cancer, the differentiated type was more prevalent in AGC compared with SGC ($P<0.01$).

A total of 457 among the 1727 cases of SGC had undergone gastric screening within 5 years before their presentation with symptoms, and 440 of them were judged as negative for malignancy (25.5% of SGC). The remaining 17 cases did not undergo the recommended close examination after the initial screening and the compliance rate was 96.3%. Of the 440 cases, 337 (76.6%) were screened with barium meal study, 54 (12.3%) with endoscopy and the remaining 49 (11.1%) with both. Nearly half of these cases (210/440) underwent screening within a year. Only 56 cases of SGC (3.2%), however, had regularly undergone annual screening. Of the 419 with AGC, 196 were either diagnosed on first screening or had not been screened in the 5 years before the screening at which the gastric tumour was detected, and the other 223 had been screened within the previous 5 years. Of the latter cases, 220 were judged to be negative for malignancy at the previous screenings (52.5% of AGC). The remaining three cases dropped out and the compliance rate was 98.7%. Of the 220 cases, 161 (73.2%) underwent screening within a year and a total of 145 cases of AGC (34.6%) had regularly undergone annual screening, 27 were screened between 1 and 2 years ago, 16 between 2 and 3 years ago, nine between 3 and 4 years ago and seven between 4 and 5 years previously. Of the 220 screened cases, 167 (75.9%) wee screened with a barium meal study, 30 (13.6%) with endoscopy and the remaining 23 (10.5%) with both. There were no differences in the pathological stage of the tumour between the cases screened within a year and those screened in the period of 1–2 years before in both SGC and AGC. Comparisons of the pathological stage according to the screening interval were done between three groups (A, cases screened within 2 years; B, screened between 2 and 5 years earlier; and C, screened more than 5 years earlier or not screened) in both AGC and SGC cases (Table III). The cases that dropped out from screening were included in group C. As for the SGC cases, there were significant differences in the pathological stages between groups A and C ($P<0.0001$) and between groups B and C ($P<0.05$). There was also a significant difference in the incidence of stage I tumours between groups A and B ($P<0.05$). In the AGC cases, there were differences in the incidence of group I tumours between groups A and C ($P<0.001$) and between B and C ($P<0.05$). This difference was not significant between groups A and B, probably because of the small sample sizes. Interestingly, there were also evident differences in the disease stages between the previously screened SGC and AGC in the similar screening interval groups.

In the 48 cases of AGC detected in advanced stages (stages III or IV), only nine cases had undergone gastric screening test within a year. In other words, 39 cases with these advanced AGC had demonstrated the gastric tumour at their initial screening or at that with an interval of at least 1 year.

A surgical curative resection was accomplished on 407 (97.1%) cases of AGC. Six of them died within the first 30 post-operative days and 11 patients were lost to the follow-up. In the following 390 patients, only 13 (3.3%) died of recurrent disease and three of them died more than 5 years after primary gastrectomy. Seventeen patients died of other (non-gastric) malignancies, while 19 died of either non-neoplastic disease or accidents. On the other hand, 1289 (74.6%) cases of SGC were curatively resected and 1171 of them were followed up. In these cases, 220 (18.8%) cases died of recurrence and 26 of them died more than 5 years after surgery. Twenty-seven died of other malignancy and 180 died of either non-neoplastic diseases or accidents. The cumulative 5 and 10 year survival rates for patients with curatively resected AGC were 85.2% and 72.2%, respectively, while those for patients with SGC were 66.8% and 55.4% (Figure 1).

### Discussion

We consider the Japanese superiority regarding the results of gastric cancer treatment compared with western countries to be attributable to the high incidence of early gastric cancer and to the adoption of radical surgery with lymphadenectomy (Asao, 1990; Okamura *et al.*, 1988; Wanebo *et al.*, 1993). As for the type of surgical intervention, the effect of extended lymph node dissection on survival remains somewhat controversial, especially in western countries (Dent *et al.*, 1988; Robertson, 1994). In Japan, a randomised study comparing conservative with more radical surgery is difficult to perform, because radical surgery is the gold standard for gastric cancer, except for patients with severe systemic

**Figure 1** The survival curves for curatively resected asymptomatic gastric cancer (A, $n=407$) and symptomatic gastric cancer (B, $n=1289$). A statistically significant difference was observed in the survival rate ($P<0.01$).

**Table III** Correlations between screening interval and pathological stage of the disease (%)

| *Screening interval* | *No. of cases* | *Stage I* | *Stage II* | *Stage III* | *Stage IV* |
|---|---|---|---|---|---|
| SGC | | | | | |
| Group A (within 2 years) | 320 (18.5) | 178 (55.6) | 22 (6.9) | 67 (20.9) | 53 (16.6) |
| Group B (2–5 years) | 120 (6.9) | 52 (43.3) | 10 (8.3) | 28 (23.3) | 30 (25.0) |
| Group C (more than 5 years or not screened) | 1287 (74.5) | 395 (30.7) | 121 (9.4) | 384 (29.8) | 387 (30.1) |
| AGC | | | | | |
| Group A | 188 (44.9) | 165 (87.2) | 10 (5.3) | 10 (5.3) | 3 (1.6) |
| Group B | 32 (7.6) | 24 (75.0) | 1 (3.1) | 6 (18.8) | 1 (3.1) |
| Group C | 199 (47.5) | 147 (73.9) | 24 (12.1) | 23 (11.6) | 5 (2.5) |

complications or rather small early cancer, which is a good candidate for conservative surgery. Early detection is unquestionably effective for improving survival (Green et al., 1988). The high incidence of gastric cancer in Japan has led to the introduction and pervasion of gastric screening and has resulted in a vigorous detection programme for gastric cancer, involving both a double-contrast barium meal study and endoscopy (Kampschoer et al., 1989).

The ultimate purpose of cancer screening is the detection of preclinical lesions (Yamazaki et al., 1989; Hisamichi and Sugawara, 1984) and, thus, asymptomatic gastric cancer is the goal of gastric cancer screening. This current study disclosed an excellent prognosis of AGC. Nearly half of all cases were detected in the period of mucosal cancer, most of the cases could be resected curatively and more than 85% of them survived over 5 years.

The optimum screening interval for detecting gastric cancer in the asymptomatic period is another problem. Shiratori et al. (1985) recommended an interval of 1.5 years of screening as being beneficial in detecting early gastric cancer. The growth rate of gastric cancer, however, is not uniform and some of them are known to show extremely rapid growth and seldom to be detected at an early stage, which thus undermines the usefulness of screening (Kodama et al., 1984; Mori and Sugimachi, 1990, Nishidoi et al., 1992). On the other hand, cases persisting in the mucosa for several years have also been reported (Adachi et al., 1990). In this study, there were evident differences in the tumour stages between SGC and AGC, in spite of a similar screening interval. We thus consider that some SGC grow more rapidly than AGC.

In the AGC cases, 48 cases were detected for a gastric tumour at stages III or IV, however, there were only nine cases detected at these advanced stages in spite of periodic annual screening. Furthermore, diffusely infiltrative cancer, including scirrhous cancer, which is known to be a rapidly growing and highly malignant tumour, was rarely found among our asymptomatic cases. This type of tumour is thus thought to develop within a few months and then rapidly progress, and annual screening alone is thus insufficient to detect it in its early phase of development (Haruma et al., 1992).

**Acknowledgement**

The authors thank Brian T Quinn for comments on the manuscript.

### References

ADACHI Y, MORI M AND SUGIMACHI K. (1990). Persistence of mucosal gastric carcinomas for 8 and 6 years in two patients. *Arch. Pathol. Lab. Med.*, **114**, 150–152.

ASAO K. (1990). A Japanese view of American early gastric cancer detection. *Gastroenterology*, **99**, 1189–1190.

DENT DM, MADDEN MV AND PRICE SK. (1988). Randomized comparison of R1 and R2 gastrectomy for gastric carcinoma. *Br. J. Surg.*, **75**, 110–112.

EDDY DM. (1983). Finding cancer in asymptomatic people. *Cancer*, **51**, 2441–2445.

GREEN PHR, O'TOOLE KM, SLONIM D, WANG T AND WEG A. (1988). Increasing incidence and excellent survival of patients with early gastric cancer: experience in a United States medical center. *Am. J. Med.*, **85**, 658–661.

HARUMA K, YOSHIHARA M, TANAKA S, SUMII K, KAJIYAMA G, HIDAKA T, DAITOKU K AND MATSUMOTO T. (1992). Rapid growth and difficulty of early detection of scirrhous carcinoma of the stomach. *Am. J. Gastroenterol.*, **87**, 31–36.

HISAMICHI S AND SUGAWARA N. (1984). Mass screening for gastric cancer by X-ray examination. *Jpn. J. Clin. Oncol.*, **14**, 211–223.

JAPANESE RESEARCH SOCIETY FOR GASTRIC CANCER. (1981). The general rules for the gastric cancer study in surgery and pathology. Part I. Clinical classification. *Jpn. J. Surg.*, **11**, 127–139. Part II. Histological classification of gastric cancer. *Jpn. J. Surg.*, **11**, 140–145.

KAMPSCHOER GHM, FUJII A AND MASUDA T. (1989). Gastric cancer detected by mass survey: comparison between mass survey and outpatient detection. *Scand. J. Gastroenterol.*, **24**, 813–817.

KODAMA Y, INOKUCHI K, KAMEGAWA T, OKAMURA T, MATSUURA K, ENJOJI M, NAKAMURA Y AND KUSABA I. (1984). Growth patterns of gastric carcinoma detected by mass survey. *Jpn. J. Surg.*, **14**, 366–370.

MORI M AND SUGIMACHI K. (1990). Clinicopathologic studies of gastric carcinoma. *Semin. Surg. Oncol.*, **6**, 19–72.

MORI M, ADACHI Y, NAKAMURA K, KUROIWA S, ENJOJI M AND SUGIMACHI K. (1990). Advanced gastric carcinoma stimulating early gastric carcinoma. *Cancer*, **65**, 1033–1040.

NISHIDOI H, KIMURA O, MAKINO M, SUGEZAWA A AND KAIBARA N. (1992). Clinicopathological features of advanced gastric cancer detected by periodic mass survey. *Jpn. J. Surg.*, **22**, 120–123.

OKAMURA T, TSUJITANI S, KORENAGA D, HARAGUCHI M, BABA H, HIRAMOTO Y AND SUGIMACHI K. (1988). Lymphadenectomy for cure in patients with early gastric cancer and lymph node metastasis. *Am. J. Surg.*, **155**, 29–33.

ROBERTSON CS, CHUNG SCS, WOODS SDS, GRIFFIN SM, RAIMES SM, LAU JTF AND LI AKC. (1994). A prospective randomized trial comparing R1 subtotal gastrectomy with R3 total gastrectomy for antral cancer. *Ann. Surg.*, **220**, 176–182.

SHIRATORI Y, NAKAGAWA S, KIKUCHI A, ISHII M, UENO M, MIYASHITA T, SAKURAI T, NEGAMI J, SUZUKI T AND SATO I. (1985). Significance of a gastric mass screening survey. *Am. J. Gastroenterol.*, **80**, 831–834.

YAMAGATA S AND HISAMICHI S. (1979). Epidemiology of cancer of the stomach. *World J. Surg.*, **3**, 663–669.

YAMAZAKI H, OSHIMA A, MURAKAMI R, ENDOH S AND UBUKATA T. (1989). A long-term follow-up study of patients with gastric cancer detected by mass screening. *Cancer*, **63**, 613–617.

WANEBO HJ, KENNEDY BJ, CHMIEL J, STEELE G, WINCHESTER D AND OSTEEN R. (1993). Cancer of the stomach: a patient care study by the American college of surgeons. *Ann. Surg.*, **218**, 583–592.